# Genome-Wide Analysis of *HIPP* Genes and Functional Analysis of *GsHIPP79* in Response to Alkaline Stress in *Glycine soja*

**DOI:** 10.3390/plants15060850

**Published:** 2026-03-10

**Authors:** Chengbo Zhang, Zichun Wei, Deqiang Ding, Zaib_un Nisa, Xiaoxia Jin, Chao Chen

**Affiliations:** 1 Department of Chemistry and Molecular Biology, School of Life Science and Technology, Harbin Normal University, Harbin 150025, China; zcblss@126.com (C.Z.); wzcfyywzy@163.com (Z.W.); dingdeqiang980227@163.com (D.D.); xiaoxia6195@126.com (X.J.); 2 General Botany Lab, Institute of Molecular Biology and Biotechnology, The University of Lahore (Defence Road Campus), Lahore 54000, Pakistan; zaib.nisa@imbb.uol.edu.pk; 3 Engineering Research Center of Agricultural Microbiology Technology (Ministry of Education), Heilongjiang University, Harbin 150500, China

**Keywords:** heavy metal-associated isoprenylated plant proteins, alkaline, *Glycine soja*, GsHIPP79

## Abstract

Heavy metal-associated isoprenylated plant protein (HIPP) family genes are known to be involved in plant development and stress responses. Even though the HIPPs have been identified and characterized in some plants, the roles of these genes in plant abiotic stress tolerance remain unclear in *G. soja* (*Glycine soja*), especially in response to alkaline stress. In the present study, a total of 79 potential *HIPP* family genes were obtained in *G. soja* using the Hidden Markov Model. Bioinformatics analysis was used to explore their physicochemical properties, gene structure, phylogenetic relationships, *cis*-acting elements, chromosomal location and collinearity. Expression profiling showed that 18 *HIPP* family genes were displayed significantly different transcript levels under alkaline stress, among which *GsHIPP79* was selected for functional characterization. The results showed that *GsHIPP79* exhibited enhanced alkaline stress tolerance in transgenic *Arabidopsis* plants, as evidenced by it exhibiting higher chlorophyll contents, strengthening the antioxidant defense system, and regulating the expression of stress-responsive marker genes. Moreover, overexpression of *GsHIPP79* in transgenic soybean hairy roots conferred enhanced alkaline stress tolerance. In conclusion, this study provided valuable information on *HIPP* family genes in *G. soja* and identified the positive roles of *GsHIPP79* in response to alkaline stress tolerance.

## 1. Introduction

Soil alkalization is a major environmental factor limiting global agricultural productivity. Approximately 950 million hectares of land worldwide are affected by salinization, a significant portion of which consists of saline–alkaline soils [1,2]. Alkali stress not only elevates soil pH but also induces a series of detrimental effects in plants, including ion toxicity, osmotic stress, and nutritional imbalance, which ultimately inhibits plant growth and reduces crop yield [3,4,5]. However, plants have developed complex molecular mechanisms to cope with alkali stress, including the regulation of ion homeostasis, scavenging of reactive oxygen species, accumulation of osmoregulatory substances, and expression regulation of stress-related genes [6,7,8].

Heavy metal-associated isoprenylated plant proteins (HIPPs) are a specific protein family characterized by the presence of a heavy metal-associated (HMA) domain and a C-terminal isoprenylation motif [9,10,11,12]. The HMA domain binds Cu^2+^, Cd^2+^, and Zn^2+^, critical for heavy metal metabolism. The C-terminal CaaX motif mediates subcellular localization, stability, and protein interactions via isoprenylation [13].

Accumulating evidence indicates that *HIPP* family members are involved not only in heavy metal transport and detoxification but also in plant responses to various abiotic stresses [14,15]. For instance, *AtHIPP33* in *Arabidopsis* enhances plant resistance to cadmium stress [16]. *OsHIPP17* regulates rice copper toxicity tolerance by modulating the expression of copper transporter and cytokinin signaling genes [17]. *HIPP29* suppresses aluminum tolerance by enhancing root aluminum accumulation in soybean [18]. Furthermore, studies have confirmed that some *HIPP* members participate in responses to cold and drought stresses [19,20]. However, the specific biological functions of *HIPP* family genes, particularly *GsHIPP* from *G. soja*, in conferring alkali stress tolerance remain poorly understood. Therefore, we hypothesized in this study that *GsHIPP* family genes may improve alkaline stress tolerance in *G. soja*.

*G. soja* displayed high tolerance to various abiotic stresses such as cadmium, aluminum, drought and salt–alkaline stresses [21,22]. In our previous studies, a highly adaptable alkaline tolerance *G. soja* line was identified, which exhibited a healthy growth under 50 mM NaHCO_3_. Further, some differentially expressed *HIPP* genes were selected in the *G. soja* line using transcriptome analysis under alkaline stress [23]. In this study, the *HIPP* family genes were identified in *G. soja*. Their physicochemical properties, gene structure, phylogenetic relationships, chromosomal location and collinearity were also investigated. Then, the *GsHIPP79* gene was selected based on its expression level in transcriptome sequencing data to identify its function under alkaline stress. The results demonstrated that *GsHIPP79* improved alkaline stress tolerance by increasing chlorophyll contents, promoting reactive oxygen species scavenging and activating the transcription of stress-responsive genes.

## 2. Results

### 2.1. Identification of GsHIPP Family Genes

To identify the *G. soja HIPP* genes, the amino acid sequences of HIPP family protein sequences from *Arabidopsis* and rice were downloaded from the EnsemblPlants database as query sequences. A total of 165 HIPP candidate amino acid sequences were identified using the Hidden Markov Model (HMM). Then, the redundant or incomplete domain sequences were removed based on Pfam and SMART databases. As a result, 79 potential *HIPP* family genes were obtained in *G. soja*, and named (*GsHIPP1* to *GsHIPP79*) based on the chromosomal location (Table 1).

The GsHIPP family proteins ranged in length from 113 (*GsHIPP33* and *GsHIPP65*) to 560 (*GsHIPP37*), amino acid residues and molecular weight (MW) from 12.97424 (*GsHIPP*33) to 60.38506 (*GsHIPP37*), and 4.88 (*GsHIPP11*) to 10.05 (*GsHIPP52*) theoretical isoelectric points (pI) values. It was also found that the aliphatic index of *GsHIPP* genes ranged from 34.66 (*GsHIPP37*) to 91.79 (*GsHIPP63*), which reflects the relative content of aliphatic amino acids. The average hydrophilicity (GRAVY value) varied from −0.249 (*GsHIPP32*) to −1.288 (*GsHIPP34*), with the negative value confirming its hydrophilic nature.

### 2.2. Phylogenetic Analysis of GsHIPP Family Genes

**Figure 1:**
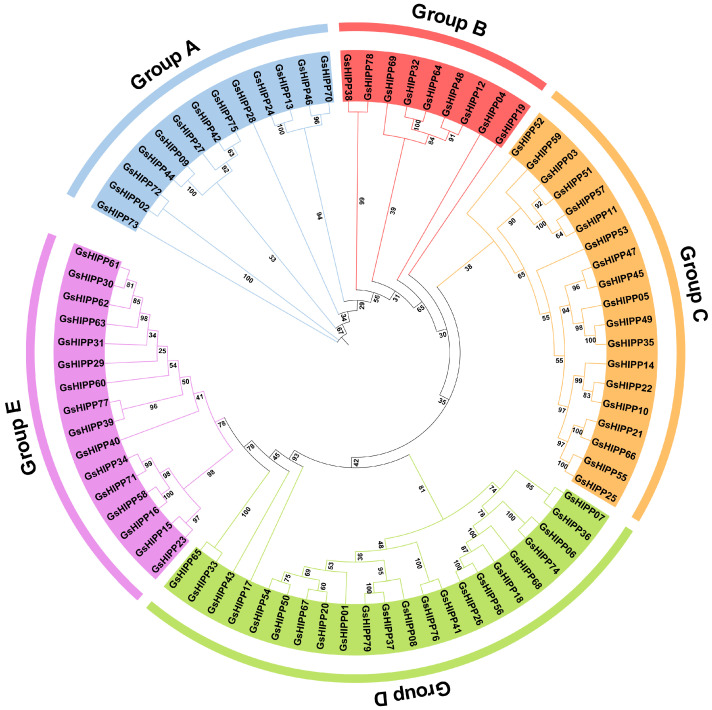
Phylogenetic tree of HIPPs in *G. soja*. The neighbor-joining (NJ) phylogenetic tree was constructed using MEGA 11 software. The reliability of the phylogenetic tree was assessed with 1000 bootstrap replications.

To explore the evolutionary relationship of *HIPP* genes, a comparative phylogenetic tree was constructed with the HIPP protein sequences from *G. soja*, *Arabidopsis* and rice. The results showed that all genes were resolved into five major groups (Figure S1). This demonstrated that the *HIPP* genes maintained evolutionary conservation across *G. soja*, *Arabidopsis*, and rice, despite these species belonging to different evolutionary lineages. Phylogenetic analysis also identified a total of 79 GsHIPP proteins in *G. soja*, which were categorized into five distinct groups (Figure 1). Groups A to E have 13, 9, 19, 22, and 16 members, respectively. Each group has a relatively distant evolutionary relationship, indicating a degree of sequence diversity within the family and functional differentiation.

### 2.3. Conserved Motif and Domain Analysis

**Figure 2:**
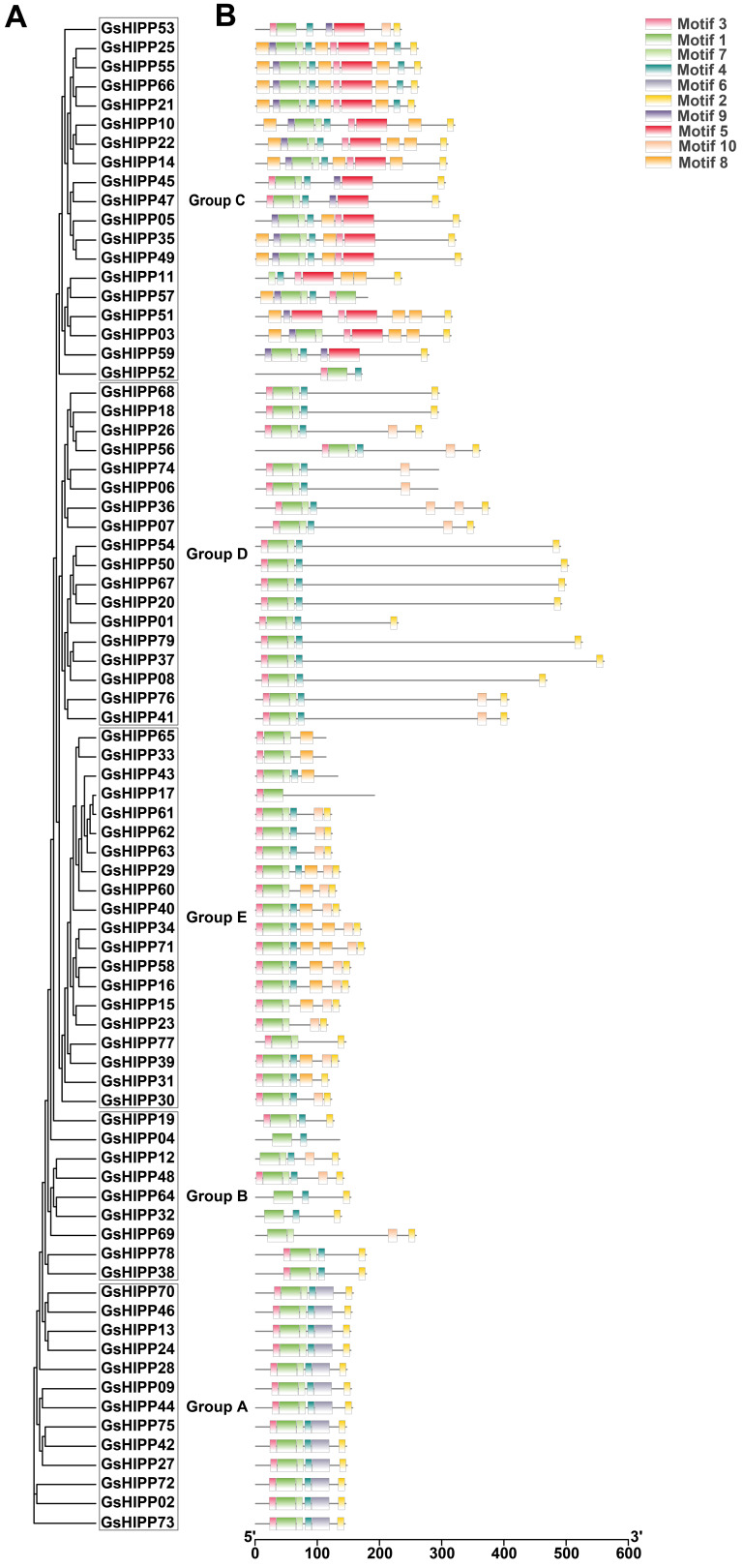
GsHIPP protein phylogeny and conserved domain analysis. (**A**) The neighbor-joining phylogenetic tree was constructed with 1000 bootstrap replications using MEGA 11 software. (**B**) The online tool MEME was employed to predict conserved motifs of GsHIPP proteins. Conserved motifs were visualized as color-coded, numbered boxes and integrated with the phylogenetic tree using TBtools-II v2.210.

MEME (Motif-based Sequence Analysis Tools) motif analysis revealed that *GsHIPP* family proteins shared ten conserved motif sequences (Motif 1 to Motif 10) (Figure 2). Most proteins contain conserved motifs 1, 2, 3, 4 and motif 7. The distribution patterns and sequence composition of these motifs were highly conserved among family members, with each motif likely serving specific functions [9]. Among them, Motif 1 belongs to the heavy metal-associated domain. Motif 2 belongs to the C-terminal isoprenylation motif. The sequence characteristics of Motif 7 matched the typical structure of a protein–protein interaction module.

### 2.4. Chromosomal Localization and Synteny Analysis

**Figure 3:**
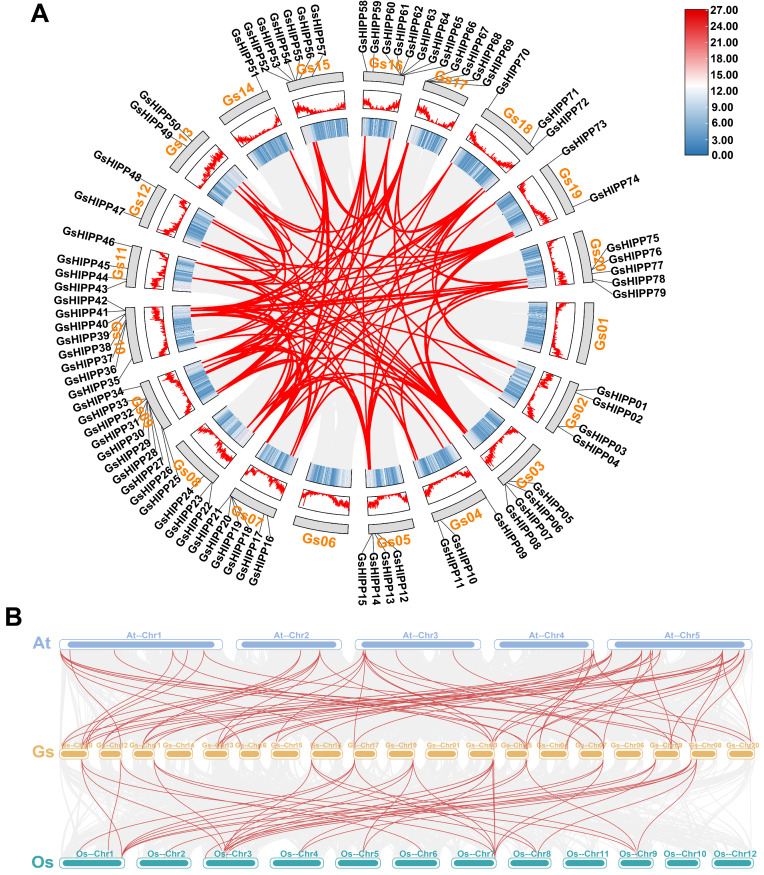
Chromosome locations and syntenic analysis of *GsHIPP* genes. (**A**) The chromosomes are represented as circles using the TBtools-II v2.210 software. Red lines linked duplicated gene pairs. (**B**) Cross-species synteny analysis among *G. soja*, *Arabidopsis*, and rice. Gray background lines represent all synteny modules; red lines indicate collinearity of *HIPP* gene pairs across species. Synteny analysis was performed with TBtools-II v2.210 software.

To explore the chromosomal localization and potential evolution mechanisms of 79 *GsHIPP* family genes, the distribution characteristics were determined using the intra-species synteny analysis. The result showed that all 79 *GsHIPP* family genes were found unevenly distributed among 18 chromosomes, with no apparent preference for clustering on specific chromosomes (Figure 3A). In addition, a total of 125 pairs of syntenic paralogs were identified, indicating that *GsHIPP* family genes exhibited whole-genome duplication or segmental duplication. Remarkably, chromosome 9 contained the highest number of segmental duplications, suggesting its crucial role in the expansion of the *HIPP* family genes. Furthermore, gene synteny maps were generated by selecting the *HIPP* families of model plants *Arabidopsis* and rice as reference genomes (Figure 3B). The analysis revealed 62 homologous pairs between *GsHIPP* and *AtHIPP* genes, and 42 homologous pairs between *GsHIPP* and *OsHIPP* genes, indicating that *GsHIPP* genes are evolutionarily more closely related to *AtHIPP* genes.

### 2.5. Analysis of Promoter Cis-Acting Elements

**Figure 4:**
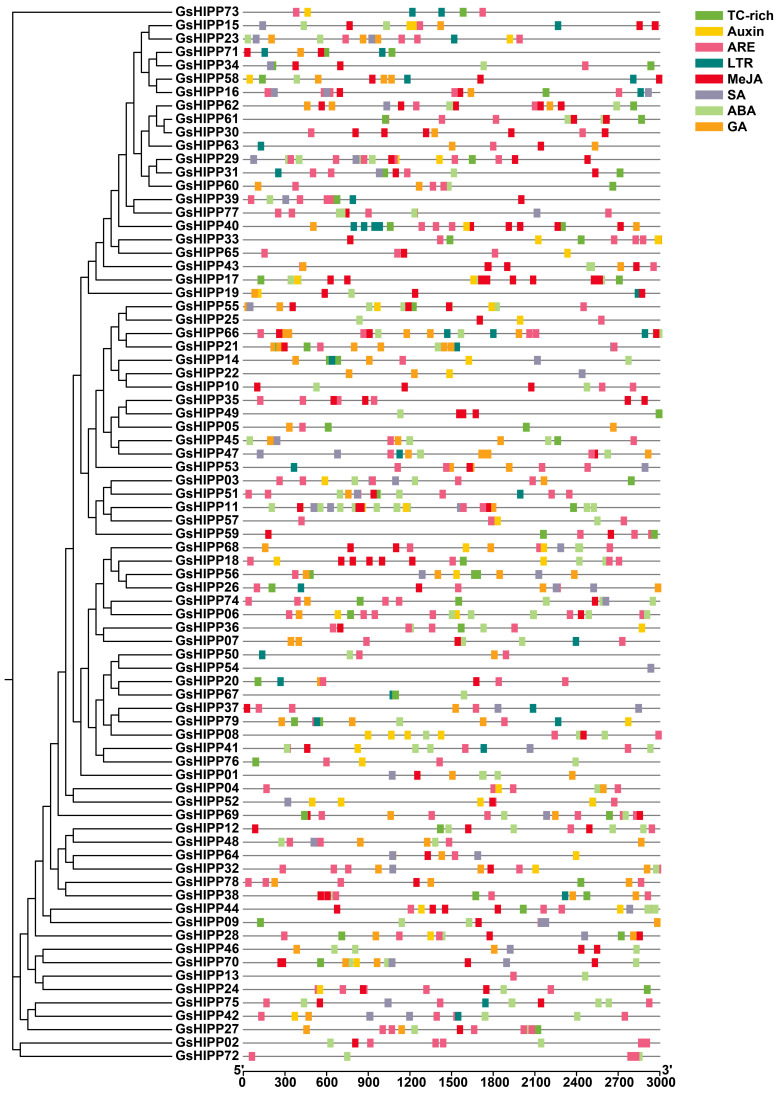
Distribution of *cis*-acting elements in the promoter of the *GsHIPP* genes. A 3000 bp upstream sequence from the start codon of each *GsHIPP* family member was extracted, and *cis*-acting elements were predicted using PlantCARE online tool. Predicted elements were visualized as color-coded, numbered boxes and integrated with the phylogenetic tree using TBtools.

To further elucidate the potential regulatory roles of *GsHIPP* genes in response to various stresses, a systematic analysis of the *cis*-acting elements within their promoter region was conducted. The 3000 bp region upstream of the start codon was predicted to investigate the *cis*-acting elements using the PlantCARE online tool. The results showed that *GsHIPP* family genes mainly contained various putative hormone-related *cis*-acting elements, including methyl jasmonate (MeJA), abscisic acid (ABA), gibberellin (GA), salicylic acid (SA) and auxin-responsive elements (Figure 4). The putative abiotic stress-responsive elements were also identified, such as defense and stress responsiveness (TC-rich), low-temperature responsiveness (LTR) and anaerobic-responsive elements (ARE). Taken together, the promoter analysis provides a molecular basis for the potential involvement of *GsHIPP* genes in hormone-regulated and stress-responsive pathways, warranting further functional and expression analyses.

### 2.6. Expression Profiling Under Alkaline Stress

**Figure 5:**
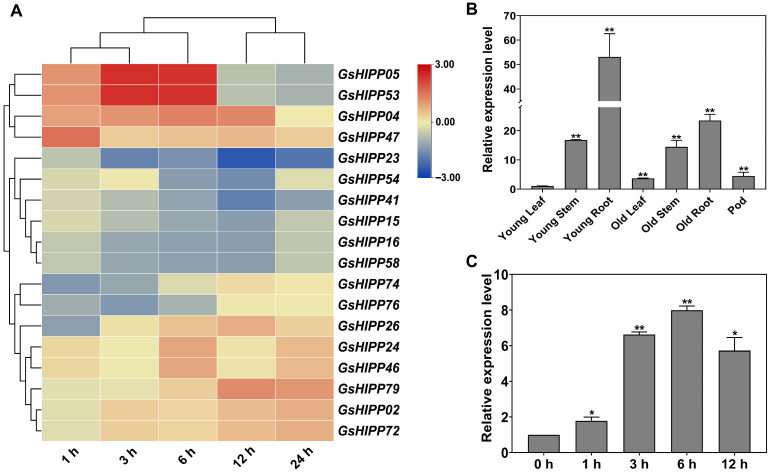
Expression patterns of the *GsHIPP* gene family and expression analysis of *GsHIPP79* under alkaline stress. (**A**) Expression patterns were derived from transcriptome data under alkaline stress. The heat map was generated using TBtools-II v2.210 software. Red and blue indicate high and low expression levels, respectively (|Log2 fold change| > 1.5, *p* < 0.05). (**B**) Expression analysis of *GsHIPP79* gene in various tissues of *G. soja*, including the seedling stage (young root, young stem and young leaf) and the mature stage (old root, old stem, old leaf and pod). (**C**) The expression of *GsHIPP79* in *G. soja* roots was assessed following treatment with 50 mM NaHCO_3_ for 0, 1, 3, 6, 12, and 24 h. Three biological replicates were analyzed via the 2^−ΔΔCt^ method using Student’s *t*-test. Statistical analyses were performed with SPSS 21.0. Asterisks in the figure denote statistical significance between groups (*: *p* < 0.05; **: *p* < 0.01).

To explore the potential roles of *G. soja HIPP* family genes in the defense responses towards alkaline stress, the expression levels were detected using transcriptome sequencing under alkaline stress [23]. The heat map showed that 18 *HIPP* family genes displayed different transcript levels (|Log2 fold change| > 1.5, *p* < 0.05) (Figure 5A). Among them, eleven *HIPP* family genes displayed up-regulated expression, while seven genes displayed down-regulated expression. These results indicated that the *G. soja HIPP* family genes may be involved in responses to alkaline stress; however, their specific roles in conferring alkali stress tolerance remain to be elucidated. Among the genes up-regulated under alkaline stress, we further randomly selected *GsHIPP79* for further investigation.

To elucidate the biological function of *GsHIPP79*, this study systematically analyzed its tissue-specific expression patterns and dynamic expression profiles under alkali stress using qRT-PCR analysis. The results showed that the *GsHIPP79* gene was differentially expressed in all tissues in *G. soja*. However, the young root exhibited higher expression (53-fold) than other tissues (Figure 5B), suggesting its potential functional importance in this organ. The expression patterns of *GsHIPP79* were further explored in roots at different time points. Under 50 mM NaHCO_3_ treatment, *GsHIPP79* expression was rapidly up-regulated at 3 h (6.5-fold) and reached a maximum value at the 6 h time point (8-fold) (Figure 5C). Collectively, these findings provide evidence that *GsHIPP79* is highly responsive to alkali stress in roots, supporting its candidacy for further functional analysis in alkali stress tolerance.

### 2.7. GsHIPP79 Enhanced Alkaline Tolerance in Arabidopsis

**Figure 6:**
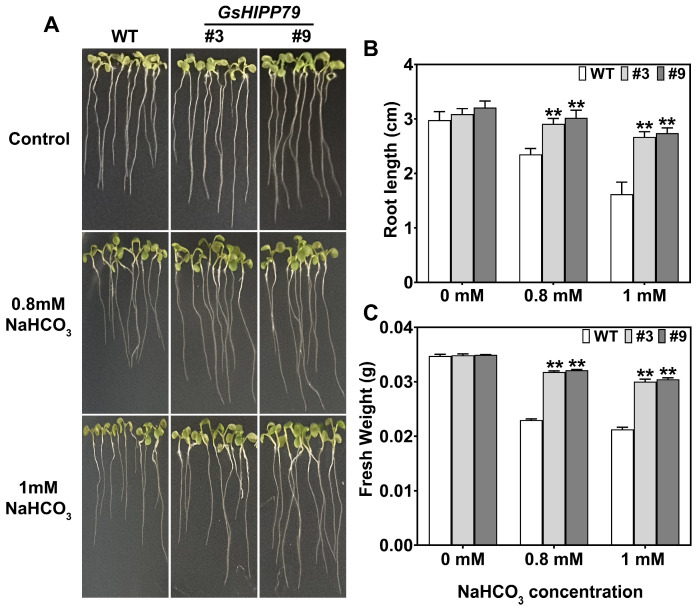
Overexpression of *GsHIPP79* enhanced tolerance under NaHCO_3_ stress in *Arabidopsis*. (**A**) Phenotype of *GsHIPP79* transgenic lines (#3 and #9) and WT grown on 1/2-strength MS medium with the addition of 0.8 and 1 mM NaHCO_3_. (**B**) Root length and (**C**) fresh weight were determined under both control and alkaline stress conditions. Data were analyzed by one-way ANOVA and Duncan’s multiple range test (*p* < 0.05). Statistical analyses were performed with SPSS 21.0. Asterisks in the figure denote statistical significance between groups (**: *p* < 0.01).

To evaluate the function of *GsHIPP79* in plant alkali stress response, we conducted phenotypic analysis of wild-type (WT) and two *GsHIPP79* overexpression lines (#3 and #9) under NaHCO_3_ (0.8 and1.0 mM) treatment. Under normal growth conditions, no significant differences in growth parameters were observed among the lines, such as root length and fresh weight. However, under NaHCO_3_ stress, although all plants exhibited growth inhibition, the transgenic lines were significantly less affected than the WT plants (Figure 6A).

Under alkaline stress, the transgenic lines of *GsHIPP79* exhibited significant advantages in both root length and fresh weight (Figure 6B,C). Further analysis revealed that the degree of growth inhibition was positively correlated with NaHCO_3_ concentration. Under different stress concentrations, the growth inhibition rate of transgenic lines was 25–40% lower than that of the WT. These results demonstrated that *GsHIPP79* enhanced plant adaptation to alkaline stress.

### 2.8. GsHIPP79 Enhanced Chlorophyll Content and Antioxidant Enzyme Activities

**Figure 7:**
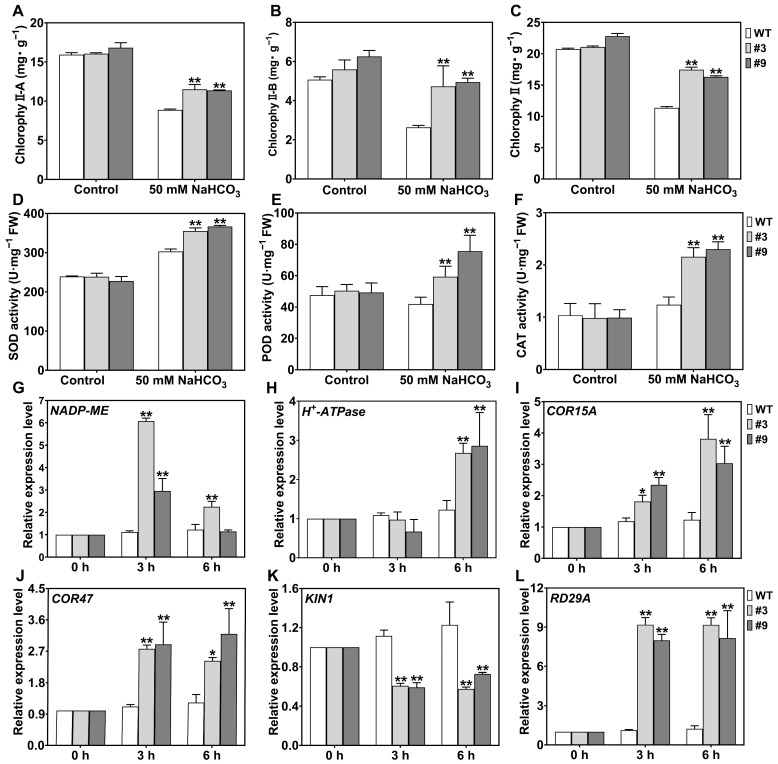
Physiological indices of *GsHIPP79* and its regulation of stress-related marker gene expression under NaHCO_3_ stress treatment. (**A**) Chlorophyll a, (**B**) chlorophyll b, and (**C**) total chlorophyll content analyses under 50 mM NaHCO_3_ stress. (**D**) SOD, (**E**) POD, and (**F**) CAT enzyme activity analyses under 50 mM NaHCO_3_ stress. Data were analyzed by one-way ANOVA and Duncan’s multiple range test (*p* < 0.05). (**G**) *NADP-ME*, (**H**) *H^+^-ATPase*, (**I**) *COR15A*, (**J**) *COR47*, (**K**) *KIN1* and (**L**) *RD29A* were determined under 50 mM NaHCO_3_ stress. Three biological replicates were analyzed via the 2^−ΔΔCt^ method using Student’s *t*-test. Statistical analyses were performed with SPSS 21.0. Different numbers of asterisks (*) in the figure indicate the level of significance between different treatment groups (*: *p* < 0.05, **: *p* < 0.01).

To further elucidate the roles of *GsHIPP79* in response to alkali stress tolerance, the chlorophyll content and antioxidant enzyme activities were analyzed in WT and transgenic *Arabidopsis* plants. Under non-stress conditions, no significant differences were observed in chlorophyll content or antioxidant enzyme activities among the different lines. However, under NaHCO_3_ stress, the transgenic lines exhibited 21%, 17%, and 18% higher chlorophyll a (Figure 7A), chlorophyll b (Figure 7B), and total chlorophyll contents (Figure 7C), respectively, compared to the WT. These results indicated that *GsHIPP79* transgenic lines mitigated the degradation of photosynthetic pigments caused by alkali stress. Moreover, the activities of superoxide dismutase (SOD) (Figure 7D), peroxidase (POD) (Figure 7E), and catalase (CAT) (Figure 7F) in transgenic plants were significantly higher than those in WT. These results suggested that *GsHIPP79* transgenic lines enhanced the scavenging capacity for reactive oxygen species (ROS) by coordinately activating multiple antioxidant enzymes, thereby alleviating oxidative damage and improving plant adaptation to alkaline environments.

### 2.9. GsHIPP79 Promoted the Expression of Stress-Responsive Gene Under Alkaline Treatment

The mechanism of *GsHIPP79*-mediated alkali stress response was investigated by analyzing the expression levels of six stress-related marker genes (*NADP-ME*, *H^+^-ATPase*, *COR15A*, *COR47*, *KIN1* and *RD29A*) in WT and transgenic lines under NaHCO_3_ treatment (Figure 7G–L). The quantitative real-time PCR (qRT-PCR) results revealed that the expression levels of *NADP-ME*, *H^+^-ATPase*, *COR15A*, *COR47* and *RD29A* were significantly higher in transgenic plants than WT, except for *KIN1*. Specifically, *NADP-ME*, *COR15A*, *COR47*, and *RD29A* all exhibited significant up-regulation at 3 and 6 h under NaHCO_3_ treatment. The expression of the *H^+^-ATPase* was not significantly induced at 3 h, whereas its transcript levels were significantly increased at 6 h. These results indicated that *GsHIPP79* activated the transcription of stress-responsive genes, with a particularly sustained promoting effect on typical stress marker genes such as *RD29A* and *COR47*, as well as metabolism-related genes like *NADP-ME*.

### 2.10. GsHIPP79 Enhanced Alkaline Tolerance in Hairy Roots of Soybean

**Figure 8:**
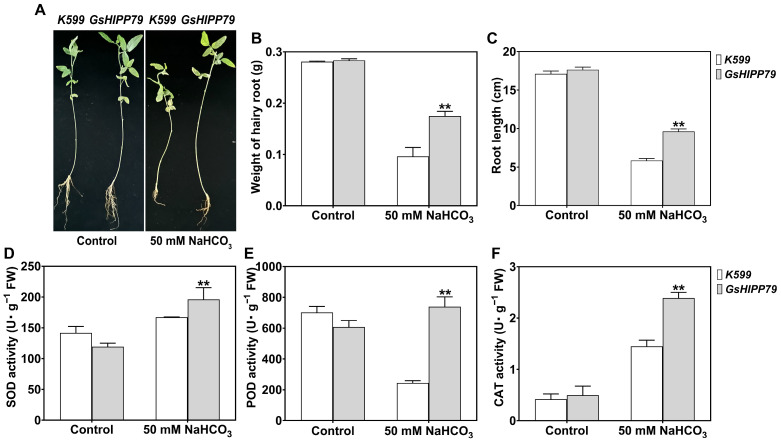
Overexpression of *GsHIPP79* enhanced tolerance under NaHCO_3_ stress in soybean. (**A**) Phenotype of *GsHIPP79* transgenic soybean hairy roots and non-transgenic (K599 control) grown on vermiculite with the addition of 50 mM NaHCO_3_. (**B**) Weight of hairy roots, (**C**) root lengths were determined under both control and alkaline stress conditions. (**D**) SOD, (**E**) POD, and (**F**) CAT enzyme activity analyses under 50 mM NaHCO_3_ stress. Data were analyzed by one-way ANOVA and Duncan’s multiple range test (*p* < 0.05). Statistical analyses were performed with SPSS 21.0. Different numbers of asterisks (*) in the figure indicate the level of significance between different treatment groups (**: *p* < 0.01).

To further confirm the roles of *GsHIPP79* in response to alkaline stress in soybean, the transgenic soybean hairy roots were obtained using the K599-mediated *Agrobacterium rhizogenes*-mediated system. Under non-stress conditions, no significant phenotypic differences were observed between *GsHIPP79* transgenic hairy roots and K599 control. Under alkaline stress treatment, the growth of all hairy roots was markedly inhibited (Figure 8A); however, the *GsHIPP79* transgenic hairy roots were significantly less inhibited than K599 control with longer root lengths and greater fresh weight (Figure 8B,C). This result was consistent with the phenotype observed in the *Arabidopsis* transgenic lines, further confirming the positive function of *GsHIPP79* in response to alkaline stress.

To validate the function of *GsHIPP79* transgenic soybean hairy roots under alkaline stress, the activities of the antioxidant enzymes were also identified in the soybean hairy root system. Under non-stress conditions, no significant differences in SOD, POD, or CAT activities were detected between transgenic hairy roots and K599 control. However, under alkaline stress, the activities of all three antioxidant enzymes were significantly higher in transgenic hairy roots than K599 control. The activities of SOD, POD and CAT increased to 1.1-fold, 3.5-fold, and 1.4-fold of control, respectively (Figure 8D–F). These findings align with previous results from the *Arabidopsis* transgenic system, indicating that *GsHIPP79* can enhance ROS scavenging capacity by coordinately activating antioxidant enzymes, thereby alleviating oxidative damage induced by alkaline stress.

## 3. Discussion

Heavy metal-associated isoprenylated plant proteins (HIPPs) are involved plant various abiotic stresses [24]. Additionally, some species of *HIPP* family genes have been identified throughout the genomes, including lotus, triticeae species and sweet cherry [19,25,26]. However, the *G. soja HIPP* family genes have not yet been identified, especially the roles of these genes in conferring alkali stress tolerance. In the present study, the HIPP family genes in *G. soja* were identified and characterized via bioinformatics analysis. Further, *GsHIPP79* was selected based on its expression level in transcriptome sequencing data and identified the positive roles under alkaline stress.

Previous studies have indicated that the *HIPP* families exhibit evolutionary divergence [27]. In this study, multiple lines of evidence also support the divergent evolution of *GsHIPP* families. Firstly, 79 potential *HIPP* family genes were identified in the *G. soja* genome. The *GsHIPP* family gene numbers is higher than in other species, such as soybean, tea and maize [11,18,28], indicating the potential gene duplication and complexity of the genome in *G. soja*. Secondly, consistent with the HIPPs in soybean [18], GsHIPP proteins varied markedly in amino acid residues, MW, pI and aliphatic index (Table 1), indicating the divergent evolution of *GsHIPP* genes in *G. soja* genome. Thirdly, the conserved motif analysis further demonstrated substantial divergence among different groups. For instance, Motif 6 and Motif 5 were exclusively found in group A and group C subfamily genes, respectively (Figure 2). The genome of palaeopolyploid soybean experienced high duplication events around 59 and 13 million years ago [29]. A total of 125 segmental duplicated gene pairs were identified across multiple chromosomes (Figure 3A), suggesting whole-genome or segmental duplication events. This result also aligned with prior studies indicating significant expansion of *HIPP* genes [11].

Nevertheless, despite the evolutionary divergence of the *GsHIPP* family genes, the groups remain relatively conserved. For example, previous studies revealed that *HIPP* family genes have been classified into five groups [11,28]. In accordance with the other species, *G. soja HIPP* family genes can be divided into five groups (Figure 1). Most subfamilies share similar conserved domains (Figure 2). The analysis revealed 62 homologous gene pairs between *GsHIPP* and *AtHIPP*, as well as 42 homologous pairs between *GsHIPP* and *OsHIPP* genes (Figure 3B). This result was also consistent with previous studies that *G. max* HIPPs are evolutionarily closely related to *AtHIPP* and *OsHIPP* genes [18], indicating the conservation of the *HIPP* genes in both *G. max* and *G. soja*.

On the other hand, the evolutionary divergence may contribute to the potential functional diversity of *GsHIPP* family genes. *Cis*-acting regulatory elements in promoter regions are involved in various elements, such as abiotic stresses and hormone-related [30]. Previous studies showed that *HIPP* genes contained various *cis*-acting elements and play important roles in response to various stresses, such as abscisic acid-related drought stress responses [31], Cd, osmotic, salt or cold stress [20,24]. In this study, *GsHIPP* family genes also contained various putative hormone-related or abiotic stress-responsive elements (Figure 4), indicating their different potential roles in response to various stresses. In addition, *GsHIPP* genes display distinct expression patterns under alkaline stress. Among them, eleven *HIPP* family genes displayed up-regulation in response to alkaline stress, while seven genes displayed down-regulated expression (Figure 5A).

To explore the functional roles of the *GsHIPP* family genes under alkaline stress, the *GsHIPP79* gene with up-regulated gene expression was selected for further validation. The expression levels of *GsHIPP79* were significantly induced under alkali stress, with the most prominent expression in the roots (Figure 5B,C). This suggests a key role for the *GsHIPP79* in root stress perception and signal transduction [17]. Overexpression of *GsHIPP79* in both *Arabidopsis* and soybean hairy roots demonstrated a significant enhancement of plant tolerance to alkaline stress (Figure 6 and Figure 8). The increased biomass and root length further verified the critical role of *GsHIPP79* in roots under alkaline stress (Figure 6B and Figure 8B,C).

Alkaline stress can activate the antioxidant defense system in plant cells [32]. As expected, *GsHIPP79* reduces oxidative damage under alkaline stress by activating the antioxidant enzymes (SOD, POD) and catalase (CAT) (Figure 7D–F and Figure 8D–F), consistent with the role of HIPPs in redox regulation [33]. Previous studies showed that *H^+^-ATPase* and *NADP-ME* contribute to salt and alkaline tolerance [34,35]. In addition, *KINI*, COR15A, COR47, and RD29A participate in responses to diverse abiotic stresses, including cold, salt or drought stress [36]. In this study, the qRT-PCR results also revealed that the expression levels of *NADP-ME*, *H^+^-ATPase*, *COR15A*, *COR47* and *RD29A* were significantly induced in transgenic plants (Figure 7G–J,L), indicating that *GsHIPP79* activated the transcription of stress-responsive genes, with a particularly sustained promoting effect on typical stress marker genes.

## 4. Conclusions

Taken together, in this study, the *HIPP* family genes were identified in *G. soja* and their physicochemical properties, gene structure, phylogenetic relationships, chromosomal location and collinearity were investigated. The positive roles of *GsHIPP79* in response to alkaline stress were confirmed by exhibiting higher chlorophyll contents, promoting reactive oxygen species scavenging and activating the transcription of stress-responsive genes. However, the mechanism underlying *GsHIPP79*’s involvement in the alkaline stress signaling pathway and its regulatory function remains unclear. Further investigations should specify the roles of *GsHIPP79* in alkaline signal transduction pathways, as well as to identify GsHIPP79-interacting proteins. Moreover, the roles of other *GsHIPP* family genes in response to alkaline stress remain to be elucidated.

## 5. Materials and Methods

### 5.1. Identification of GsHIPP Family Genes in G. soja Genome

Based on the information of *HIPP* family genes in the literature [37], the amino acid sequences of HIPP family proteins from *Arabidopsis* and rice were downloaded from the EnsemblPlants database (https://plants.ensembl.org/index.html accessed on 12 March 2024). All sequences were aligned using Clustal Omega [38]. A HMM was subsequently constructed from the aligned sequences to identify members of the *GsHIPP* family [39]. The model (HMM profile 2.3.2) was then applied to search the *G*. *soja* genome database, yielding candidate sequences. After filtering low-matching-rate sequences, *GsHIPP* candidates were screened via Pfam and SMART databases to exclude overlapping or incomplete domain sequences. Gene IDs and amino acid residues were sourced from Phytozome (https://phytozome-next.jgi.doe.gov/ accessed on 25 March 2024). The physicochemical properties of the *GsHIPP* proteins, including molecular weight and theoretical isoelectric point, were predicted using TBtools-II v2.210 software [40].

### 5.2. Bioinformatics Analysis of GsHIPP Family Genes

The NJ phylogenetic tree was constructed using MEGA 11 software. The reliability of the phylogenetic tree was assessed with 1000 bootstrap replications [41]. Chromosomal localization information for *GsHIPP* family members was extracted from the *G*. *soja* genome annotation file and visualized using TBtools-II v2.210 software. Synteny analysis was performed with TBtools-II v2.210 software. Whole-genome alignments were conducted to identify syntenic blocks between *G*. *soja* and *Arabidopsis* as well as rice [42].

The online tool MEME (Multiple EM for Motif Elicitation, http://meme-suite.org/ accessed on 8 April 2024) was employed to predict conserved motifs of GsHIPP proteins. The parameters were set as follows: the maximum number of motifs to predict was 10, and the motif length was limited to a range of 6 to 50 amino acids. Iterative and statistical analyses were used to identify statistically significant conserved short peptide modules [43].

A 3000 bp sequence upstream of the *GsHIPP* family members start codon was extracted as the promoter region. The PlantCARE online tool (http://bioinformatics.psb.ugent.be/webtools/plantcare/html/ accessed on 20 April 2024) was used to predict *cis*-acting elements within this region. The results were visualized using TBtools-II v2.210 software [44].

The expression patterns of the *GsHIPP* family genes were downloaded based on transcriptome data under alkaline stress [23]. The heat map was generated using TBtools-II v2.210 software.

### 5.3. Expression Analysis of GsHIPP79

The wild soybean seeds (G07256) were treated with concentrated sulfuric acid for 12 min with shaking. Then the seeds were rinsed six times with sterile distilled water and transferred to wet filter paper in plates for two days to promote germination. The germinated seedlings were then transplanted into growth boxes filled with Hoagland’s nutrient solution [45]. The wild soybean seedlings were grown in a plant light incubator (Xinyi, Shanghai, China) under conditions of 22–28 °C temperature, 70% relative humidity, and a 16 h light/8 h dark cycle. For alkali stress treatment, 21-day-old seedlings were exposed to Hoagland nutrient solution supplemented with 50 mM NaHCO_3_. Root samples were collected at six time points (0, 1, 3, 6, 12 and 24 h) post-stress initiation, with three biological replicates per time point. To investigate the expression patterns in different tissues, samples were collected from roots, stems, and leaves of seedlings at 21 days old (hydroponically grown), as well as from roots, stems, leaves, and pods of mature plants at three months old (soil-grown). Three biological replicates were collected for each tissue. Each biological replicate was performed with three technical replicates. Total RNA was extracted from roots using a plant total RNA extraction kit (Omega Bio-Tek, Beijing, China), followed by cDNA synthesis via a reverse transcription kit (Thermo Fisher, Waltham, MA, USA) and qRT-PCR analysis.

### 5.4. Phenotypic Analysis of GsHIPP79 in Arabidopsis and Soybean Hairy Roots Under Alkali Stress

Total RNA was extracted from leaves of *G. soja* seedlings using a plant total RNA extraction kit (Omega Bio-Tek, Beijing, China), followed by cDNA synthesis via a reverse transcription kit (Thermo Fisher, Waltham, MA, USA). Gene-specific primers for *GsHIPP79* (listed in Table S1) were designed to amplify the full-length CDS. The CDS of *GsHIPP79* was cloned into the pCAMBIA230035S vector. Then, the recombinant pCAMBIA230035S:*GsHIPP79* vector was introduced into *Agrobacterium tumefaciens* strain GV3101, and transgenic *Arabidopsis* plants were generated via the floral dip method [46]. The *Arabidopsis* plants were grown in a greenhouse under conditions of 19–21 °C, 60% relative humidity, and a 16 h light/8 h dark cycle. Homozygous seeds were selected on 1/2-strength MS medium supplemented with 25 mg L^−1^ kanamycin. The T_3_ transgenic lines #3 and #9 were randomly selected for phenotype analysis (Figure S2A). For the root length and fresh weight assays, *Arabidopsis* seeds of transgenic lines and WT were planted on 1/2-strength MS medium. After five days of incubation, seedlings exhibiting uniform growth were transferred to 1/2-strength MS medium supplemented with 0.8 mM or 1.0 mM NaHCO_3_ treatment. Fresh weight and root length were measured after two weeks.

The cultivated soybean cultivar Dongnong 50 was used as the plant material. The soybean seedlings were grown in a plant light incubator (Xinyi, Shanghai, China) under conditions of 22–28 °C temperature, 95% relative humidity, and a 16 h light/8 h dark cycle. The five-day-old soybean seedlings were inoculated with *Agrobacterium rhizogenes* K599 harboring pCAMBIA230035S:*GsHIPP79* (Figure S2B). Once soybean hairy roots reached 3–4 cm, plants were transferred to vermiculite and irrigated with 50 mM NaHCO_3_ solution. The fresh weight and root length of soybean hairy roots were measured after two weeks. Transgenic soybean hairy roots were obtained following the established protocol [47].

For analysis of physiological indicators, seeds of WT and transgenic lines (#3 and #9) were sown in a 1:1 soil and vermiculite mixture. The *Arabidopsis* plants were grown in a greenhouse under conditions of 19–21 °C, 60% relative humidity, and a 16 h light/8 h dark cycle. After 50 mM NaHCO_3_ treatment, *Arabidopsis* leaves from the control and stress groups were collected. The soybean hairy roots were collected after two weeks under 50 mM NaHCO_3_ treatment. Each 0.1 g sample was placed in a tube, flash-frozen in liquid nitrogen and stored at −80 °C. Chlorophyll content was determined using the spectrophotometric method [48]. POD activity was measured following the guaiacol method [49]. SOD activity was assessed using the nitroblue tetrazolium method [50]. CAT activity was quantified via the ultraviolet absorption method [51]. Data was analyzed by one-way ANOVA and Duncan’s multiple range test (*p* < 0.05). Statistical analyses were performed with SPSS 21.0, and data visualization was done using GraphPad Prism 8.0.1.

### 5.5. qRT-PCR Analysis

Seeds of WT and transgenic *Arabidopsis* lines (#3 and #9) were grown on 1/2 MS medium for 12 days. Uniformly sized seedlings were selected and transferred to 50 mM NaHCO_3_ stress treatment. Seedlings were collected at 0, 3, and 6 h post-treatment. Total RNA was extracted from roots using a plant total RNA extraction kit (Omega Bio-Tek, Beijing, China), followed by cDNA synthesis via a reverse transcription kit. The qRT-PCR was performed using the 7500 Real-Time PCR system and UltraSYBR Mix (BaiaolaiboBiotech, Beijing, China). Data was normalized against reference genes (*GAPDH* in *G. soja* and *Actin2* in *Arabidopsis*). Primer sequences are listed in Table S1. Three biological replicates were analyzed via the 2^−ΔΔCt^ method using Student’s *t*-test. Statistical analyses were performed with SPSS 21.0, and data visualization was done using GraphPad Prism 8.0.1.

## Figures and Tables

**Table 1:** Protein information of *GsHIPP* family genes in *G. soja*.

Gene Name	Gene ID	Amino Acid Residues	MW (kDa)	pI	Aliphatic Index	GRAVY Value
*GsHIPP01*	*GlysoPI483463.02G060900*	229	26.35835	7.55	63.36	−0.683
*GsHIPP02*	*GlysoPI483463.02G080400*	145	16.28085	9.62	71.79	−0.408
*GsHIPP03*	*GlysoPI483463.02G184400*	314	35.54651	4.96	65.45	−0.962
*GsHIPP04*	*GlysoPI483463.02G184500*	135	15.66821	9.6	74.96	−0.474
*GsHIPP05*	*GlysoPI483463.03G146000*	329	36.37318	8.82	57.42	−1.016
*GsHIPP06*	*GlysoPI483463.03G172700*	293	31.71533	7.01	52.94	−1.013
*GsHIPP07*	*GlysoPI483463.03G195500*	352	38.6954	8.26	53.92	−0.88
*GsHIPP08*	*GlysoPI483463.03G198900*	468	51.54598	8.03	52.29	−1.017
*GsHIPP09*	*GlysoPI483463.04G003300*	154	17.10249	9.11	68.25	−0.551
*GsHIPP10*	*GlysoPI483463.04G157600*	320	36.31521	5.65	56.62	−1.102
*GsHIPP11*	*GlysoPI483463.04G184300*	235	26.16989	4.88	75.45	−0.657
*GsHIPP12*	*GlysoPI483463.05G131900*	135	15.50176	8.95	54.96	−0.673
*GsHIPP13*	*GlysoPI483463.05G153100*	153	17.12578	9.42	71.24	−0.586
*GsHIPP14*	*GlysoPI483463.05G172700*	308	35.2261	6.02	56.92	−1.226
*GsHIPP15*	*GlysoPI483463.05G179700*	136	15.49084	8.66	70.88	−0.986
*GsHIPP16*	*GlysoPI483463.07G059200*	151	17.20196	8.8	66.49	−0.988
*GsHIPP17*	*GlysoPI483463.07G087600*	191	21.31676	6.68	57.07	−0.568
*GsHIPP18*	*GlysoPI483463.07G206100*	294	31.67941	9.08	50.07	−0.859
*GsHIPP19*	*GlysoPI483463.07G210400*	126	14.45298	8.56	70.16	−0.475
*GsHIPP20*	*GlysoPI483463.07G215000*	492	52.09348	6.5	43.01	−1.104
*GsHIPP21*	*GlysoPI483463.07G224900*	257	29.49659	5.48	63.62	−1.22
*GsHIPP22*	*GlysoPI483463.08G010300*	309	35.4702	5.78	55.15	−1.277
*GsHIPP23*	*GlysoPI483463.08G016900*	116	13.14122	8.79	79.74	−0.625
*GsHIPP24*	*GlysoPI483463.08G129000*	153	17.10978	9.42	72.55	−0.568
*GsHIPP25*	*GlysoPI483463.09G012400*	261	29.9101	5.27	69.35	−1.072
*GsHIPP26*	*GlysoPI483463.09G042100*	269	29.51633	9.3	62.71	−0.704
*GsHIPP27*	*GlysoPI483463.09G093700*	147	16.491	9.58	72.86	−0.467
*GsHIPP28*	*GlysoPI483463.09G093900*	147	16.6391	5.52	70.82	−0.49
*GsHIPP29*	*GlysoPI483463.09G100100*	136	15.48111	8.97	80.81	−0.541
*GsHIPP30*	*GlysoPI483463.09G100200*	122	13.61385	8.3	84.51	−0.347
*GsHIPP31*	*GlysoPI483463.09G100500*	118	13.26459	9.02	80.08	−0.605
*GsHIPP32*	*GlysoPI483463.09G106000*	138	15.4006	6.58	70.72	−0.249
*GsHIPP33*	*GlysoPI483463.09G106100*	113	12.97424	9.22	71.5	−1.249
*GsHIPP34*	*GlysoPI483463.09G207700*	170	19.6737	6.69	59.59	−1.288
*GsHIPP35*	*GlysoPI483463.10G051100*	322	36.04835	8.28	59.63	−0.961
*GsHIPP36*	*GlysoPI483463.10G123300*	376	40.40297	8.19	60.16	−0.749
*GsHIPP37*	*GlysoPI483463.10G127100*	560	60.38506	9.07	34.66	−1.202
*GsHIPP38*	*GlysoPI483463.10G141100*	178	20.68266	9.21	67.3	−0.598
*GsHIPP39*	*GlysoPI483463.10G194000*	134	15.10233	6.59	84.25	−0.598
*GsHIPP40*	*GlysoPI483463.10G194100*	136	15.43793	8.66	82.28	−0.673
*GsHIPP41*	*GlysoPI483463.10G224400*	407	44.2932	8.35	53.29	−0.855
*GsHIPP42*	*GlysoPI483463.10G224500*	146	16.53895	9.47	65.96	−0.673
*GsHIPP43*	*GlysoPI483463.11G065600*	132	14.95347	9.11	78.18	−0.766
*GsHIPP44*	*GlysoPI483463.11G083700*	156	17.35488	9.23	69.87	−0.52
*GsHIPP45*	*GlysoPI483463.11G157600*	305	33.31283	8.76	62.26	−0.897
*GsHIPP46*	*GlysoPI483463.11G217500*	155	17.0366	9.35	72.84	−0.484
*GsHIPP47*	*GlysoPI483463.12G072700*	296	32.38891	9.03	59.53	−0.927
*GsHIPP48*	*GlysoPI483463.12G174900*	142	16.26915	7.67	56.97	−0.808
*GsHIPP49*	*GlysoPI483463.13G109600*	332	36.74012	8.45	59.31	−0.957
*GsHIPP50*	*GlysoPI483463.13G167700*	503	53.09025	8.36	40.14	−0.992
*GsHIPP51*	*GlysoPI483463.14G150100*	316	35.61452	4.96	63.48	−0.967
*GsHIPP52*	*GlysoPI483463.15G048200*	171	19.06996	10.03	71.64	−0.449
*GsHIPP53*	*GlysoPI483463.15G079600*	234	26.96009	9.26	69.36	−1.009
*GsHIPP54*	*GlysoPI483463.15G096600*	490	51.65083	8.41	38.61	−0.967
*GsHIPP55*	*GlysoPI483463.15G108700*	267	30.57572	5.13	65.62	−1.134
*GsHIPP56*	*GlysoPI483463.15G133800*	361	39.5464	8.99	69.14	−0.537
*GsHIPP57*	*GlysoPI483463.15G175300*	180	19.78007	8.48	79.06	−0.502
*GsHIPP58*	*GlysoPI483463.16G029100*	153	17.42626	8.81	68.1	−0.977
*GsHIPP59*	*GlysoPI483463.16G069700*	278	31.58651	9.18	79.17	−0.905
*GsHIPP60*	*GlysoPI483463.16G143400*	130	14.83717	7.62	77.15	−0.768
*GsHIPP61*	*GlysoPI483463.16G143500*	122	13.56281	6.95	85.33	−0.293
*GsHIPP62*	*GlysoPI483463.16G143600*	123	13.78801	8.29	76.67	−0.438
*GsHIPP63*	*GlysoPI483463.16G143700*	123	13.88837	8.76	91.79	−0.297
*GsHIPP64*	*GlysoPI483463.16G149900*	153	17.38797	7	75.88	−0.184
*GsHIPP65*	*GlysoPI483463.16G150000*	113	13.03228	9.11	70.62	−1.279
*GsHIPP66*	*GlysoPI483463.17G005600*	262	29.89703	5.57	62.79	−1.203
*GsHIPP67*	*GlysoPI483463.17G015200*	499	53.38209	7.29	44.35	−1.104
*GsHIPP68*	*GlysoPI483463.17G023500*	295	32.04064	8.91	50.24	−0.938
*GsHIPP69*	*GlysoPI483463.17G027200*	258	29.27089	8.83	63.02	−0.909
*GsHIPP70*	*GlysoPI483463.18G017900*	157	17.39081	9.17	65.1	−0.596
*GsHIPP71*	*GlysoPI483463.18G182300*	176	20.44651	6.15	57.56	−1.352
*GsHIPP72*	*GlysoPI483463.18G232300*	145	16.22281	9.6	72.48	−0.386
*GsHIPP73*	*GlysoPI483463.19G001000*	144	16.42878	9.79	68.19	−0.503
*GsHIPP74*	*GlysoPI483463.19G170900*	294	31.4489	6.21	52.41	−1.002
*GsHIPP75*	*GlysoPI483463.20G094500*	146	16.58395	9.49	65.34	−0.705
*GsHIPP76*	*GlysoPI483463.20G094600*	407	44.23212	7.64	54.74	−0.834
*GsHIPP77*	*GlysoPI483463.20G129200*	145	16.39272	5.87	87.93	−0.41
*GsHIPP78*	*GlysoPI483463.20G181100*	178	20.8038	9.32	66.74	−0.648
*GsHIPP79*	*GlysoPI483463.20G193500*	525	57.37915	8.99	34.74	−1.177

## Data Availability

The datasets generated and analyzed during the current study are available from the corresponding authors on reasonable request.

## Supplementary Materials

The following supporting information can be downloaded at: https://www.mdpi.com/article/10.3390/plants15060850/s1, Table S1: Gene-specific primers used in this study. Figure S1: Phylogenetic tree of HIPPs in *G. soja*, *Arabidopsis* and rice. The neighbor-joining (NJ) phylogenetic tree was constructed using MEGA 11 software. The reliability of the phylogenetic tree was assessed with 1000 bootstrap replications. Figure S2: Identification of *GsHIPP79* transcript levels in transgenic *Arabidopsis* and soybean hairy roots. (A) RT-PCR analysis of *GsHIPP79* expression in transgenic *Arabidopsis*. (B) qRT-PCR analysis of *GsHIPP79* expression in soybean hairy roots. Three biological replicates were analyzed via the 2^−ΔΔCt^ method using Student’s *t*-test. Statistical analyses were performed with SPSS 21.0. Asterisks in the figure denote statistical significance between groups (**: *p* < 0.01).

## Abbreviations

The following abbreviations are used in this manuscript:

HIPPs	Heavy metal-associated isoprenylated plant proteins
*G. soja*	*Glycine soja*
HMA	Heavy metal-associated
MW	Molecular weight
pI	Theoretical isoelectric points
NJ	Neighbor-Joining
MeJA	Methyl jasmonate
ABA	Abscisic acid
GA	Gibberellin
SA	Salicylic acid
TC-rich	Defense and stress responsiveness
LTR	Low-temperature responsiveness
ARE	Anaerobic responsive elements
*G. max*	*Glycine max*
WT	Wild-type
SOD	Superoxide dismutase
CAT	Catalase
POD	Peroxidase
ROS	Reactive oxygen species
qRT-PCR	Quantitative real-time PCR
HMM	Hidden Markov Model
MEME	Motif-based Sequence Analysis Tools
