# Supplementary material for: Genome-Wide Analysis of HIPP Genes and Functional Analysis of GsHIPP79 in Response to Alkaline Stress in Glycine soja
Source: Plants (Basel). 2026 Mar 10;15(6):850. doi: 10.3390/plants15060850 (PMC13029564; doi:10.3390/plants15060850)
Supplement: Supplementary file 1 [file plants-15-00850-s001.zip › Figure S1.pdf]

Supplementary Figure S1. Phylogenetic tree of HIPP proteins in *G. soja, Arabidopsis* and rice.

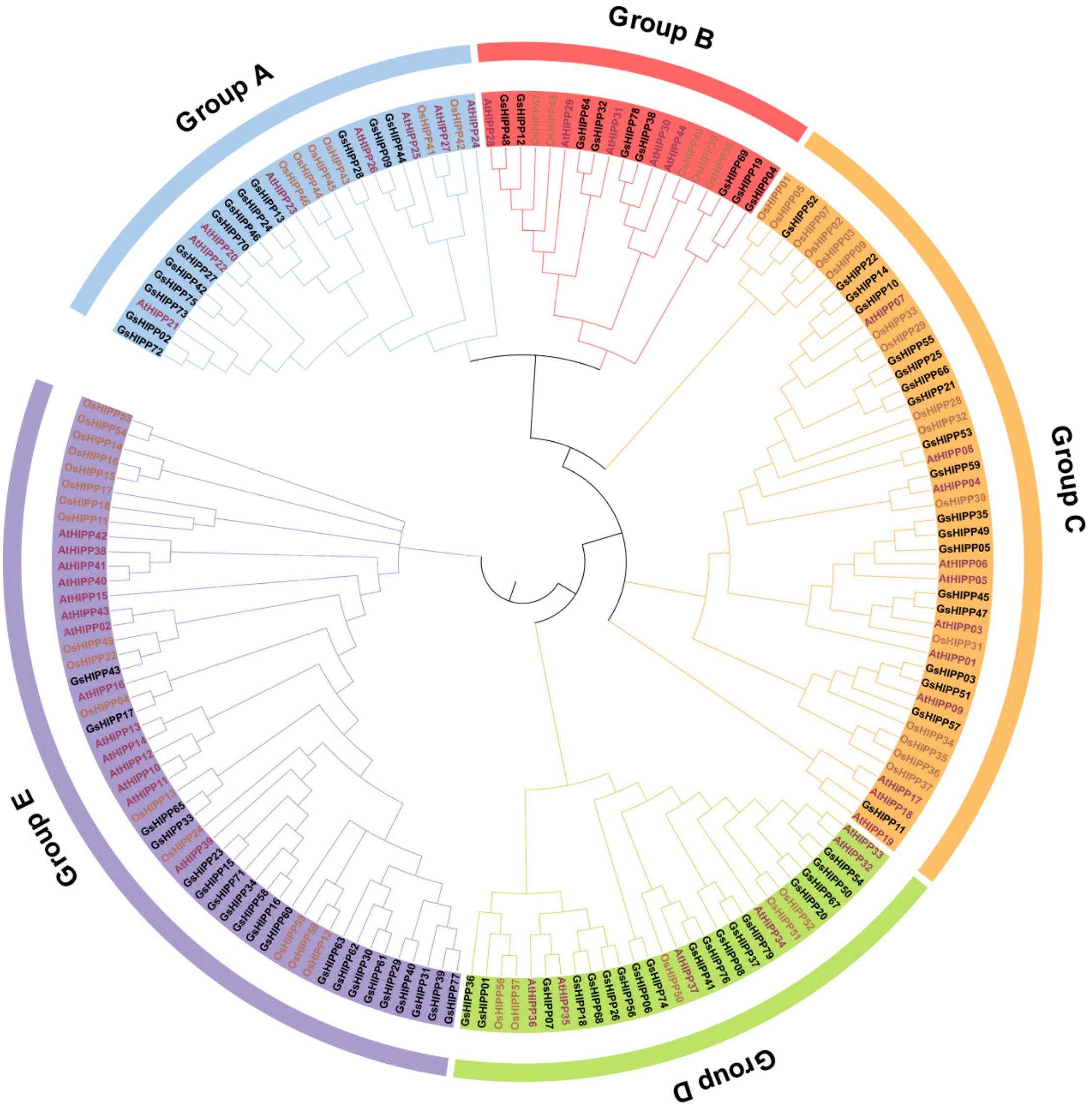

The neighbor-joining (NJ) phylogenetic tree was constructed using MEGA 11 software. The reliability of the phylogenetic tree was assessed with 1000 bootstrap replications.
