# Supplementary material for: Genome-Wide Analysis of HIPP Genes and Functional Analysis of GsHIPP79 in Response to Alkaline Stress in Glycine soja
Source: Plants (Basel). 2026 Mar 10;15(6):850. doi: 10.3390/plants15060850 (PMC13029564; doi:10.3390/plants15060850)
Supplement: Supplementary file 1 [file plants-15-00850-s001.zip › Figure S2.pdf]

Supplementary Figure S2. Identification of *GsHIPP79* transcript levels in transgenic *Arabidopsis* and soybean hairy roots.

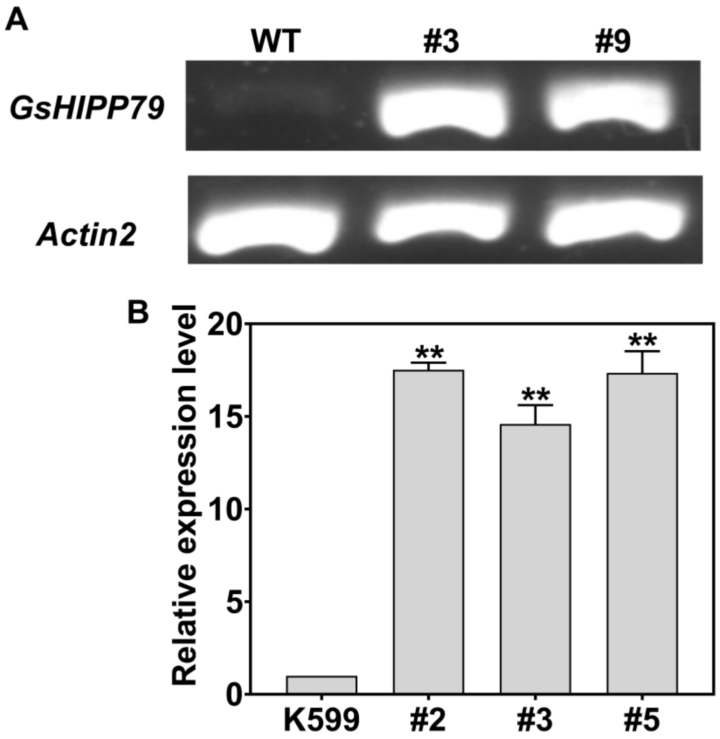

(A) RT-PCR analysis of *GsHIPP79* expression in transgenic *Arabidopsis*. **(B)** qRT-PCR analysis of *GsHIPP79* expression in soybean hairy roots. Three biological replicates were analyzed via the $2^{-\Delta\Delta Ct}$ method using Student's *t*-test. Statistical analyses were performed with SPSS 21.0. Asterisks in the figure denote statistical significance between groups (**: p <0.01).
