# Supplementary material for: Genome-Wide Analysis of HIPP Genes and Functional Analysis of GsHIPP79 in Response to Alkaline Stress in Glycine soja
Source: Plants (Basel). 2026 Mar 10;15(6):850. doi: 10.3390/plants15060850 (PMC13029564; doi:10.3390/plants15060850)
Supplement: Supplementary file 1 [file plants-15-00850-s001.zip › Table S1.pdf]

Supplementary Table S1 Gene-specific primers used in this study.

Gene ID	Primer sequence (5′-3′)
GADPH	Forward: GACTGGTATGGCATTCCGTGT
	Reverse: GCCCTCTGATTCCTCCTTGA
NADP-ME	Forward: TGGTCTGATCTACCCGCCATT
	Reverse: CGCCAATCCGAGGTCATAGG
H^+-ATPase	Forward: TTTGGATTATAAACCTCACTATATG
	Reverse: CCAGTCATTCCAACAATATGC
COR15A	Forward: AATTTCAAGCACTTAAACTCGT
	Reverse: AGAATGTGACGGTGACTGTG
COR47	Forward: GGAGTACAAGAACAACGTTCCCGA
	Reverse: TGTCGTCGCTGGTGATTCCTCT
KIN1	Forward: AACAAGAATGCCTTCCAAGC
	Reverse: CGCATCCGATACACTCTTTCC
RD29A	Forward: GGCGTAACAGGTAAACCTAGAG
	Reverse: TCCGATGTAAACGTCGTCC
GsHIPP79 (For qRT-PCR)	Forward: AACGGCCATAAAGGGAATGGG
	Reverse: GGAAGCCACCACCATTTTTGCC
GsHIPP79 (For gene clone)	Forward: CGGAATTCCCTCTACACTCTAGTCTCTCGCTG
	Reverse: CCATCGATGCACTAGGACAAAATTTAATGAAC
Actin2	Forward:TTACCCGATGGGCAAGTC
	Reverse: GCTCATACGGTCAGCGATAC
